# MicroRNA Regulation of Oxidative Stress

**DOI:** 10.1155/2017/2872156

**Published:** 2017-10-31

**Authors:** Jaideep Banerjee, Savita Khanna, Akash Bhattacharya

**Affiliations:** ^1^George Washington University, Washington, DC, USA; ^2^The Ohio State University, Columbus, OH, USA; ^3^University of Texas Health Science Center, San Antonio, TX, USA

MicroRNAs (miRNAs) as a biomarker of pathology and regulators of gene expression have been well established through numerous publications over the last 10 years. Oxidative stress-related tissue damage is an important component of many diseases, and more and more studies are focused on discovering the signature of the regulatory interactions between redox signaling and specific miRNAs attributing to the disease. Reactive oxygen species has two faces—the good ROS and the bad ROS. Sustained high levels of ROS can cause intracellular damage while oxidants are also important intracellular signaling molecules, and multiple studies over the last two decades have implicated redox-dependent signaling as essential to a host of cellular decisions including differentiation, growth, cell death, and senescence. This makes it all the more important for a well-regulated cellular ROS level, and miRNAs fill in the role of maintaining this homeostasis. A dysregulation of normal physiological miRNA levels can thus lead to oxidative damage and development of disease.

This special issue on microRNA regulation of oxidative stress encompasses cutting edge articles that focus on the role of microRNAs in regulating redox biology in several pathological conditions.

- Atherosclerosis. Endothelial cell apoptosis under oxidative stress plays a critical role in the initiation and progression of atherosclerosis. T. Li et al. have discovered the role of miRNA-210 in protecting human umbilical vein endothelial cells against oxidative stress-induced apoptosis. miRNA-210 is one of the most well-characterized hypoxamiRs. The authors discovered that overexpression of miRNA-210 inhibited apoptosis and reduced ROS level in HUVECs treated with H_2_O_2_ and downregulated caspase levels. Thus, miRNA-210 has a prosurvival and antiapoptotic effect on HUVECs under oxidative stress.
- Vascular diseases. Endothelial cell dysfunction is implicated in various vascular diseases. Intracellular exchange of miRNAs can happen through packaging and releasing through exosomes. H. Liu et al., in their article, describe a unique cross-talk between neural progenitor cells (NPCs) and endothelial cells interacting through direct physical contact or through paracrine mechanisms and illustrate that miRNA-210 is the key player in the protective effects of NPC-EXs on attenuating Ang II-induced ROS overproduction and dysfunction in endothelial cells, majorly through the Nox2/ROS and VEGF/VEGFR2 signals.
- Myocardial infarction. Cardiomyocytes are exposed to oxidative stress during ischemia/reperfusion and often undergo apoptosis. Y.-C. Fang and C.-H. Yeh explore the role of miRNAs in influencing cardiomyocyte apoptosis resulting from ischemia-induced myocardial infarction. The authors determined that miRNA-302 expression is elevated by hypoxia/reoxygenation injury, and this increase in miRNA-302 expression aggravated cardiomyocyte apoptosis by inhibiting antiapoptotic Mcl-1 expression, thereby activating proapoptotic molecules. These findings suggested that elevated miRNA-302 levels can be detrimental to cells, whereas decreased miRNA-302 levels are beneficial and can lead to an effective therapeutic intervention.
  Nrf2 and MAFG are two transcription factors shown to be highly involved in the regulation of numerous antioxidant and detoxifying genes. In form of heterodimer, the complex Nrf2:MAFG binds to and activates the transcription of antioxidant/xenobiotic genes harboring antioxidant responsive elements (ARE), located in their transcription regulatory sequences. R. Caggiano identified a new “redoximiR” and discovered that miRNA-128 directly targets MAFG and in the process influences stress responses mediated by ARE-dependent genes. This work highlights a potential new prognostic and/or diagnostic marker for skeletal muscle diseases and cardiovascular ischemic diseases.
- Hodgkin lymphomas (HL). HL is a cancer that starts in the lymphocytes. In their paper, P. Karihtala et al., for the first time, assessed the microRNAs that regulate antioxidant enzymes in HL. In a group of 41 HL patients, quantification of the miRNA levels from freshly frozen lymph node samples revealed that miRNA-23b correlated inversely with CD3 and CD20 expressions and miRNA-144 with CD3, CD20, and CD30. High MnSOD mRNA levels associated with poor HL-specific outcome in the patients with advanced disease, and high miRNA-122 levels associated with worse HL-specific survival in the whole patient population. When standardized according to the CD30 expression, high miRNA-212 and miRNA-510 levels predicted worse relapse-free survival. The authors therefore successfully identified novel biomarkers for HL.
- Type 2 diabetes mellitus (T2DM). T2DM is characterized by inflammatory and oxidant status. L. Sun et al. studied the effect of a fruit juice of *Actinidia chinensis Planch* (FJACP) on the antioxidant and anti-inflammatory status on 122 Type 2 diabetes mellitus (T2DM) patients. The authors found that the treatment resulted in higher levels of serum miRNA-424, which was positively related to Keap1 and Nrf2 levels, while Keap1 and Nrf2 levels were positively related to the levels of SOD and GSH and negatively related to proinflammatory IL-1 beta and IL-6. The authors thus concluded that FJACP treatment can be developed as a novel nonpharmaceutical intervention for T2DM patients.
- Neurological disorders (stroke and dementia). J. Song et al. investigated whether miRNA-let7A controls the damage of brain endothelial cells in a hyperglycemic state. Hyperglycemia can trigger the disruption of blood-brain barrier (BBB), leading to diverse neurological diseases including stroke and dementia. The authors found that miRNA-let7A overexpression significantly prevented cell death and loss of tight junction proteins and attenuated proinflammatory response and nitrite production in the hyperglycemic cells and thus attenuated brain endothelial cell damage. Manipulation of miRNA-let7A may therefore provide a novel solution in controlling BBB disruption which is a major precursor of central nervous system diseases.
- Cancer and aging. Hepatocellular carcinoma (HCC) is the fifth most common cancer and the third cause of cancer-related mortality worldwide. The study by Y. Wan identified miRNAs in hepatocellular carcinoma (HCC) cells which are involved in oxidative stress-response. An integrated analysis of miRNA expression signature revealed four miRNAs (miRNA-34a-5p, miRNA-1915-3p, miRNA-638, and miRNA-150-3p) elevated under oxidative stress and were found to play an important role in antiapoptosis process. The authors found that these four miRNAs were associated with patients' overall survival and thus may offer new strategies for HCC diagnosis and prognosis.
  Wen Li et al., in their work, identified a direct relationship between ULK1 and miRNA-93 and suggested that autophagy may be important for sustaining the viability of the cancerous cell line during stressful hypoxia condition.
  Cellular senescence or cell growth arrest drives the aging process and age-related disorders. Aging-induced dysregulation of miRNA biogenesis proteins is reported to promote aging and aging-associated pathologies. The comprehensive review article by H. Bu covers the importance of miRNAs in regulating oxidative stress in the context of aging and cellular senescence.
  The findings reported in the article by J. Chao et al. reveal novel mechanisms of kallistatin in protection against senescence, aging, and cancer by modulating miR-34a and miR-21 levels and inhibiting oxidative stress. Kallistatin is an endogenous protein that regulates gene signaling via its active site and the heparin-binding domain. Through its heparin-binding site, kallistatin inhibits inflammation and oxidative stress by antagonizing TNF-*α*-induced NADPH oxidase activity and miR-21 expression, NF-*κ*B activation, and inflammatory gene expression in endothelial cells. Through its active site, kallistatin inhibits miRNA-34a synthesis and stimulates eNOS and SIRT1 expressions in endothelial progenitor cells. Thus, by dual downregulation of miRNA-34a and miRNA-21 expressions, kallistatin treatment attenuates oxidative damage and cellular senescence. Also, by silencing miRNA-21, kallistatin inhibits endothelial-mesenchymal transition that is a major contributor to organ fibrosis and tumor metastasis.
- Hepatic steatosis: X.-Y. Guo studied a novel layer of epigenetic interaction between circRNA and miRNA and discovered a potential approach to the therapy of lipid peroxidative damage. Their findings reveal a circRNA-0046367/miR-34a/PPAR*α* regulatory system underlying hepatic steatosis. Expression of circRNA-0046367, which is an endogenous modulator of miR-34a, is lost during hepatocellular steatosis resulting in miR-34a's inhibitory effect on peroxisome proliferator-activated receptor *α* (PPAR*α*). PPAR*α* restoration by circRNA-0046367 normalization led to the transcriptional activation of genes associated with lipid metabolism, including carnitine palmitoyltransferase 2 (CPT2) and acyl-CoA binding domain containing 3 (ACBD3), thus ameliorating lipoxidative stress and resulting in the steatosis resolution.
- Chronic kidney disease. Renal tubulointerstitial fibrosis (TIF) is a prominent pathological feature of chronic kidney disease (CKD) leading to end-stage renal failure. Blocking or reversing TIF is one way to halt CKD progression. Y. Fang and his team studied the role of miRNA-382 in an obstructed kidney and reported that the abundance of miRNA-382 was associated with silencing of heat shock protein 60 (HSPD1) along with upregulation of 3-nitrotyrosine (3-NT) and downregulation of thioredoxin (Trx). Their work identifies a novel mechanism in which miRNA-382 contributes to the redox imbalance in the development of renal fibrosis.

Taken together, the articles in this special issue contributed by the experts in the fields of oxidative stress biology highlight the increasing importance of studying the role of redox-sensitive microRNAs to identify more effective biomarkers and develop better therapeutic targets for the plethora of oxidative stress-related diseases.

*Jaideep Banerjee* *Savita Khanna* *Akash Bhattacharya*

